# Supplementary material for: MetaRibo-Seq measures translation in microbiomes
Source: Nat Commun. 2020 Jun 29;11:3268. doi: 10.1038/s41467-020-17081-z (PMC7324362; doi:10.1038/s41467-020-17081-z)
Supplement: Supplementary file 10 — Supplementary Data 7 [file 41467_2020_17081_MOESM10_ESM.zip › File2/Confidence_High_Taxonomy/116226_out.krona.html]

Javascript must be enabled to view this page.

members
magnitude
magnitudeUnassigned
count
unassigned
taxon
rank

116226\_out

2

1

SRS893366\_contig\_number\_contig-100\_23148.23149

2
superkingdom
1

1
phylum
1239

91061
class
1

order
1
1385

186817
1
family

1386
1
genus

1
species group
86661

1
species
1396

SRS020869\_contig\_number\_1011
